# Quad-port multiservice integrated optically transparent automotive antenna for vehicular classification applications

**DOI:** 10.1038/s41598-023-44475-y

**Published:** 2023-10-17

**Authors:** Lekha Kannappan, Sandeep Kumar Palaniswamy, Malathi Kanagasabai, Jayaram Kizhekke Pakkathillam, Sachin Kumar, Mousa I. Hussein

**Affiliations:** 1 Department of Electronics and Communication Engineering, SRM Valliammai Engineering College, Kattankulathur, 603203 India; 2 Department of Electronics and Communication Engineering, SRM Institute of Science and Technology, Kattankulathur, 603203 India; 3 Department of Electronics and Communication Engineering, College of Engineering, Guindy, Anna University, Chennai, 600025 India; 4 Department of Electronics and Communication Engineering, Amrita School of Engineering, Amrita Vishwa Vidyapeetham, Chennai, 601103 India; 5 Department of Electrical Engineering, United Arab Emirates University, 15551 Al Ain, United Arab Emirates

**Keywords:** Engineering, Electrical and electronic engineering

## Abstract

The demand for vehicular antennas increases in tandem with the need for multiple features in automobiles. The development of optically transparent antenna (OTA) has made it possible to deploy antennas on delicate surfaces such as glass. Earlier studies on OTA demonstrated its viability using materials such as transparent conducting oxides (TCO) and conductive polymers. A tri-band OTA is proposed in this paper for vehicular applications. The antenna operates at 1.8 GHz, 2.4 GHz and 3.39–12 GHz bands, covering automotive/wireless applications such as GSM, Bluetooth, Wi-Fi, vehicular communication and electronic toll collection. The proposed OTA is developed on soda lime glass, and the material TCO is used for the radiator and the ground plane. The antenna prototype is tested on windshield and in an anechoic chamber, the gain and efficiency are found to be greater than 1 dBi and 80%, respectively. Furthermore, a machine learning technique for vehicle classification is proposed, which could help in electronic toll collection, automatic vehicle identifier, and parking management applications. The presented algorithm achieves 80% classification accuracy with a minimum window size.

## Introduction

For futuristic electronic communication, multiband, multiservice antennas are necessary elements. In addition to the integration of multiple bands, the applications also demand for lightweight, low-profile, inexpensive and compact sized antennas. The size of the antenna increases as multiple bands are incorporated in the design^1^. Therefore, significance has to be given to maintain balance among size, weight, and need for the applications. The multiband approach in a single antenna not only reduces the number of antennas required, it also lessens the system complexity, cost, and overall device size.

Also, area required for placing the prototype in the systems such as automobiles, satellites, mobile phones, and wearable electronics is to be considered. The antenna for automobiles can be mounted on the roof, mirror, trunk, and windshield of the vehicle^2^. The antenna developed on the transparent substrates including glass can be used on windshield. It does not affect the aesthetic appearance and technical functionality of the vehicle. A challenging part of optically transparent antenna (OTA) design is to achieve improved gain and efficiency. The OTA is developed using transparent substrates such as soda lime glass, plexiglass, Perspex, quartz, and borosilicate glass. Transparent conducting oxide (TCO) like fluorine doped tin oxide (FTO), indium doped tin oxide (ITO), silver tin oxide (AgHT), and other films like MMMC are used as conducting layer in the OTA. Decreasing the thickness of the film reduces the efficiency^3^, whereas increasing the thickness affects the transparency. The thin-film transparent conductors also include surface losses, ground losses, and skin-depth losses, which induces low efficiency and reduces the conductivity of the OTA^4^. In^5^, a polyamide substrate transparent antenna with 86% optical transmittance is proposed. The antenna resonates for a bandwidth of 5.18–5.32 GHz, and offers a peak gain of 1.9 dBi and maximum efficiency of 65.87%. Many transparent substrates are used to develop OTA, and, in^6^, cellulose acetate is used to develop the substrate with a transparent film of ITO. It provides a peak gain of 5.4 dBi and the overall size of the antenna is 50 mm × 51 mm. The graphene is used to develop the conducting film on a substrate in^7^. Graphene has low sheet resistance, which increases gain, and a good flexibility and environmental stability. The reported antenna is designed on a glass substrate with an overall size of 60 µm × 100 µm and provides an efficiency of 67%.

The transparent antenna can be developed with multilayer film to increase efficiency. In^8^, a multilayer conducting film with layers of IZTO/Ag/IZTO and polyimide substrate is used. The peak gain and efficiency of the antenna are 1.84 dBi and 54% respectively. In^9^, a high transparent antenna with optical transparency of 88% is developed with borosilicate glass substrate and ITO film. The antenna radiates for UWB with a peak gain of 3 dBi. But, the efficiency of the antenna is low ~ 27%. The fabrication of the OTA can be done using many methods like dc sputtering and chemical vapor deposition, and in^10^ the antenna is fabricated using inkjet printing. The prototype is bendable with PDMS substrate and ITO film. The antenna resonates at 2.4 GHz with 5.75 dBi. In^11^, a 300 mm × 300 mm corning glass of good transparency is used to design the antenna with AgCl film. The proposed antenna offers 60% efficiency and peak gain of 5.5 dBi. The OTA with borosilicate substrate is developed in^12^ with fluorine doped tin oxide film. The proposed antenna offers transparency greater than 50% and resonate over 5 GHz. Antenna with acryl substrate with three types of films (copper, multilayer film, multi-mesh film) is developed and compared in^13^. The study showed that the antenna developed with multilayer film (IZTO/Ag/IZTO) showed low efficiency of 7.76% and negative gain of − 4.23 dB, but transparency greater than 80%. Wired metal mesh (WMM) and micro metal mesh (µMM) are used as transparent conductive film in^14^ to fabricate radiating patch of the antenna with acrylic substrate. An optical transparency of 80% is achieved with 49% efficiency, and the overall size of the antenna is 50 mm × 50 mm. (1) In^15^, a two-port MIMO antenna is designed, where the antenna elements are placed vertically to achieve vertical polarization. In^16^, a two-port OTA is designed on soda lime glass, and the antenna elements are placed in vertical orientation with single polarization vector. In^17^, a four-port MIMO antenna is designed using AgHT-8 and plexiglass materials. The antenna operates in the frequency range of 0.69–2.82 GHz, and the antenna elements are placed horizontally to provide horizontal polarization vector. The MIMO antennas reported in the literature provides only single polarization vector and operates at single frequency.

In this work, a four-port optically transparent antenna is designed for automotive applications. The antenna is dual-polarized with tri-band resonances. The ITO coated soda lime glass substrate is chosen due to its affordability and availability, and wet chemical etching method is opted for easy fabrication. The overall size of the proposed MIMO antenna is 55 mm × 55 mm.

## Optical material properties

The OTA is developed with TCO like tin oxide, indium oxide, zinc oxide, and cadmium oxide. These oxides are further doped with dopants like fluorine, indium, gallium, aluminum, silver, and so on. These oxides conduct when doped with the appropriate dopants. For better conductivity, the optical absorptions should be less than 104/cm and optical energy gap should be more than 3 eV.

**Figure 1:**
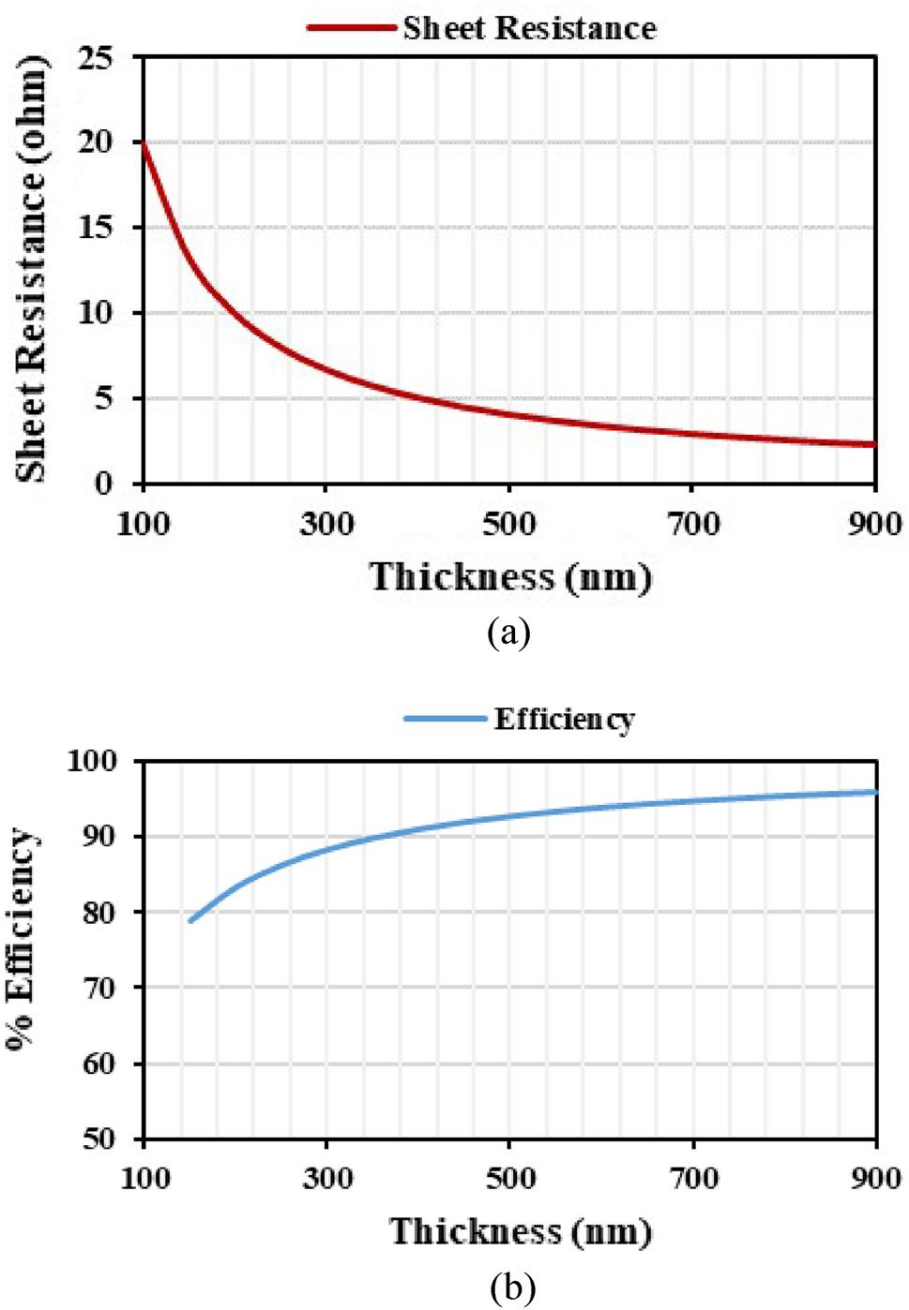
(**a**) Sheet resistance, (**b**) variation of efficiency and thickness.

The parameters that affect the efficiency of the antenna are sheet resistance and thickness. The surface resistance and conductivity are inversely proportional to each other. The surface resistance reduces when doping concentration is increased. The optical transmittance of the material is affected when the thickness is increased. But increasing the thickness increases the efficiency. So, trade-off have to be maintained among thickness, optical transmittance, and efficiency^18,19^. The surface resistance is calculated by Eq. (1). The efficiency is calculated using Eq. (2) with *R*_*a*_ as 50 Ω. The sheet resistance and efficiency of the ITO are depicted in Fig. 1.

1 $$ R_{s} = \frac{2L}{{W\sigma \delta \left( {1 - e^{{\frac{t}{2\delta }}} } \right)}}\;\;{\text{or}}\;\;R_{s} = \frac{1}{\sigma t} $$

2 $$ \% eff = \frac{{R_{a} }}{{R_{a} + R_{s} }} \times 100 $$

where *R*_*s*_ = Surface resistance, *L* = Length of the patch, *W* = Width of the patch, *t* = Thickness of the film, *δ* = Skin depth, *σ* = Conductivity of the material, *R*_*a*_ = Resistance of the antenna

**Figure 2:**
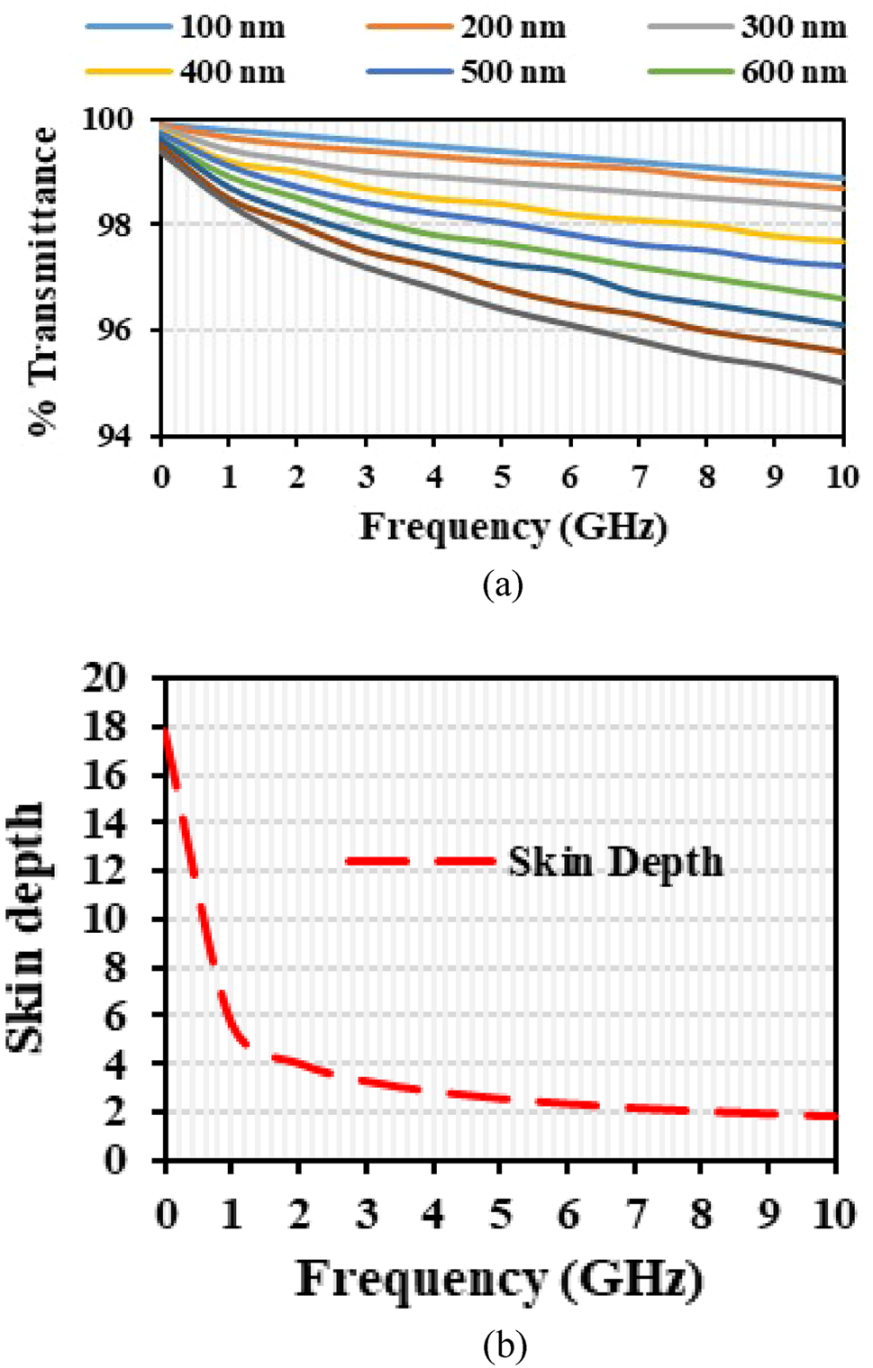
(**a**) Optical transmittance of the material, (**b**) variation of skin depth and thickness.

Using Drude’s model^20^, the optical transmittance of the ITO is analyzed using Eq. (3). Figure 2 represents the optical transmittance and skin depth of the material with respect to the frequency. Equation (4) shows the skin depth (δ) of the material.

3 $$ T\left( t \right) = e^{{ - \left( {\frac{t}{2\delta }} \right)}} $$

4 $$ \delta = \frac{{2m^{*} w^{2} \tau }}{{Z_{\infty } q^{2} N_{e} }} $$

where *Z*_*∞*_^2^ = 377^2^/*ε*_*∞*_ is the high frequency impedance for the patch antenna, the effective electron mass m^*^, electron relaxation time *τ*, *w* is the frequency, *N*_*e*_ is the electron concentration, and *q* is the charge of the electron. Therefore, the thickness of the material is chosen to be 200 nm to maintain the transparency of > 85%.

## Design of tri-band transparent antenna

### Development of single element

**Figure 3:**
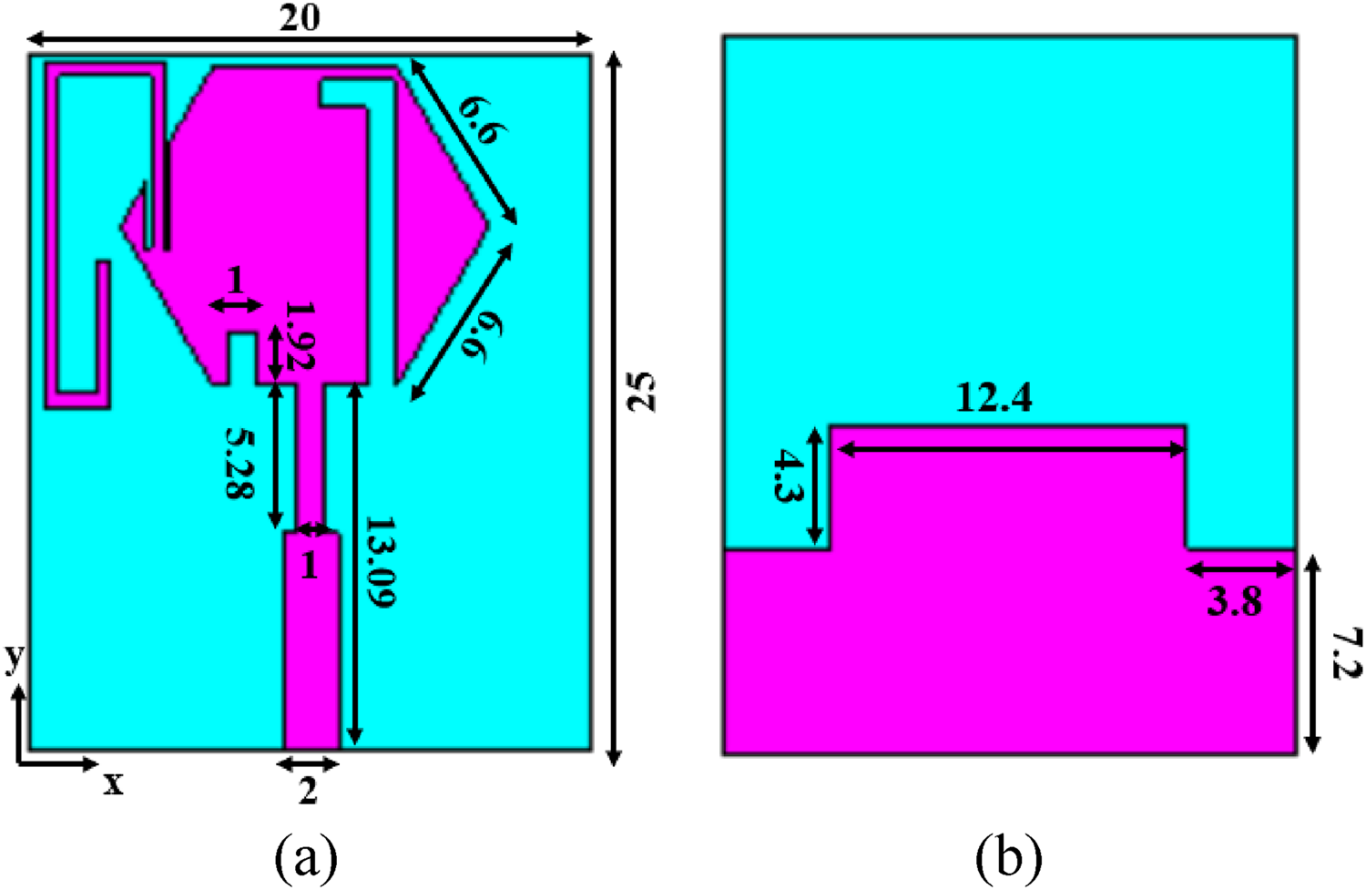
Schematic of unit cell with dimensions (mm) (**a**) radiator, (**b**) ground plane.

The geometry of the proposed OTA is shown in Fig. 3. The antenna element is developed on a transparent soda lime glass substrate of dielectric constant of 7.3, loss tangent of 0.04, and thickness of 1.1 mm. The conductive layer is developed with transparent conductive film ITO. The ITO is sputtered on the soda lime glass with a thickness of 200 nm and conductivity of 5 × 10^5^ S/m. The patch and ground are developed with ITO film. The optical transmittance of ITO is greater than 85%. The overall size of the single antenna element is 25 mm × 20 mm × 1.1 mm.

**Figure 4:**
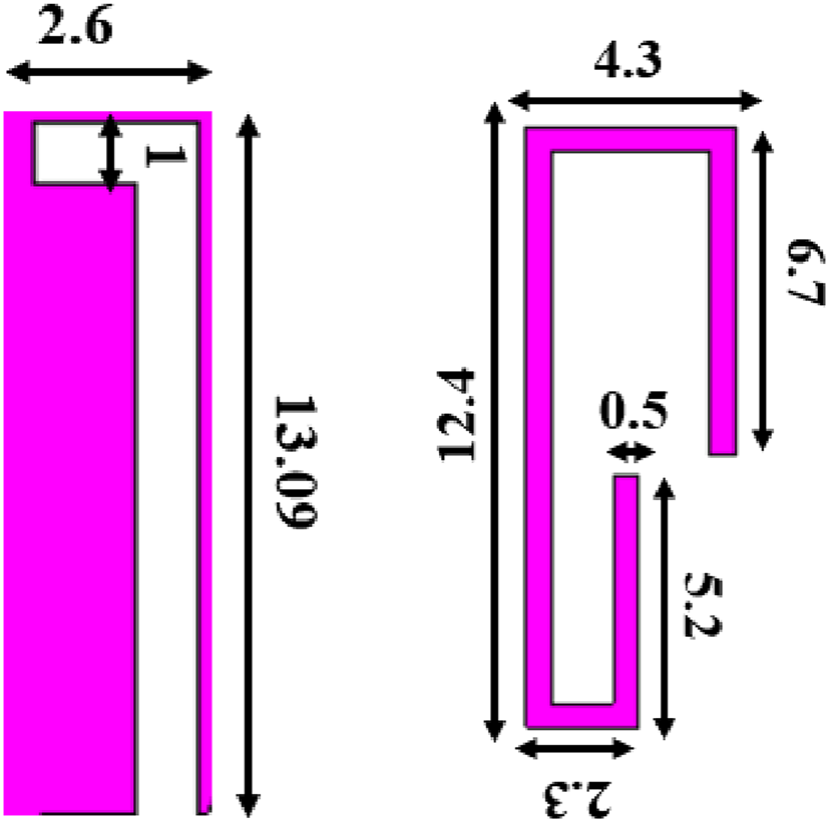
Slot and stub added for additional resonances (dimensions in mm).

**Figure 5:**
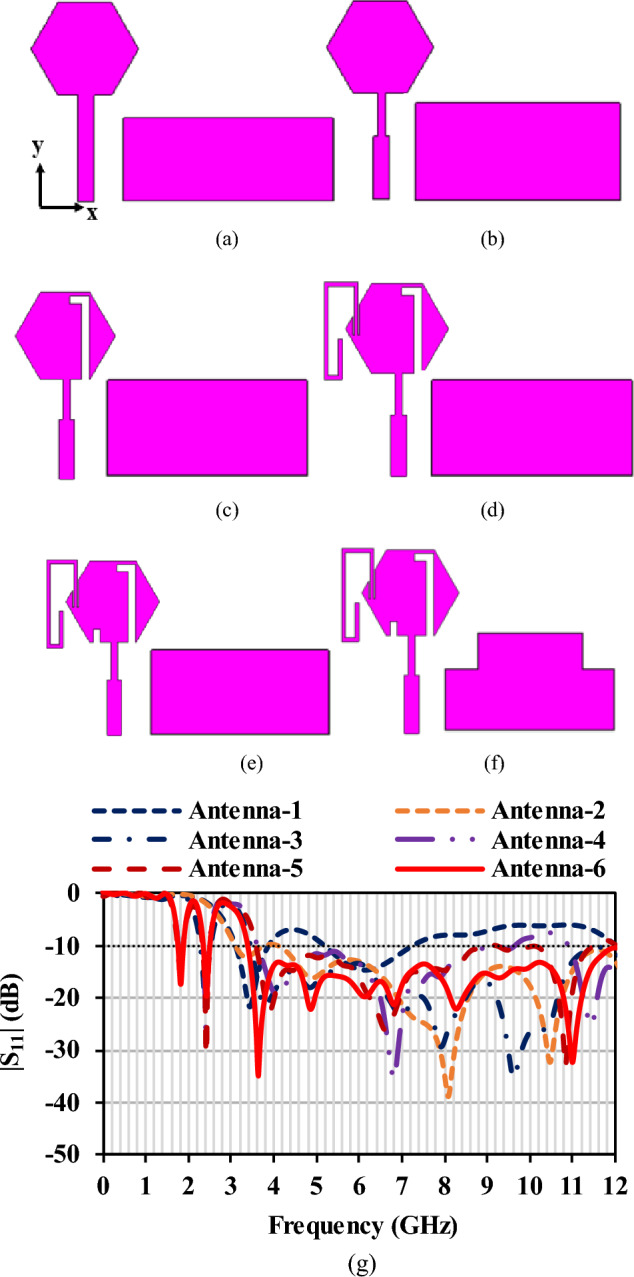
Antenna evolution (**a**) Antenna-1, (**b**) Antenna-2, (**c**) Antenna-3, (**d**) Antenna-4, (**e**) Antenna-5, (**f**) Antenna-6, (**g**) Reflection coefficients of the evolution stages.

The design starts with a basic hexagonal radiator with side of 6.6 mm. The antenna is matched to 50 Ω impedance with feed line of width 2 mm. The feed line is tapered near the radiator to increase the current near the slot and feed line, which improves the impedance matching. The hexagonal radiator with half ground plane and tapered feed results in a wideband resonance covering 3.39–12 GHz (Antenna 1 and Antenna 2). For additional resonance of 2.4 GHz, the surface current of the radiator is observed, and an inverted L-slot is introduced (Antenna 3). The 1.8 GHz is achieved by incorporating a bent stub in the radiator (Antenna 4). The added stubs create impedance mismatch over the UWB, which is further compensated by loading a slot (Antenna 5) in the radiator and a defect in the ground (Antenna 6). The inverted L-shaped slot has a resonant length of 0.11*λ*_0_, and the 1.8 GHz stub has a resonant length of 0.19*λ*_0_. Figure 4 shows the slot and stub used for achieving the additional resonances. Figure 5 shows the evolution of the antenna and its corresponding reflection coefficient characteristics.

### Four-port MIMO/diversity antenna

**Figure 6:**
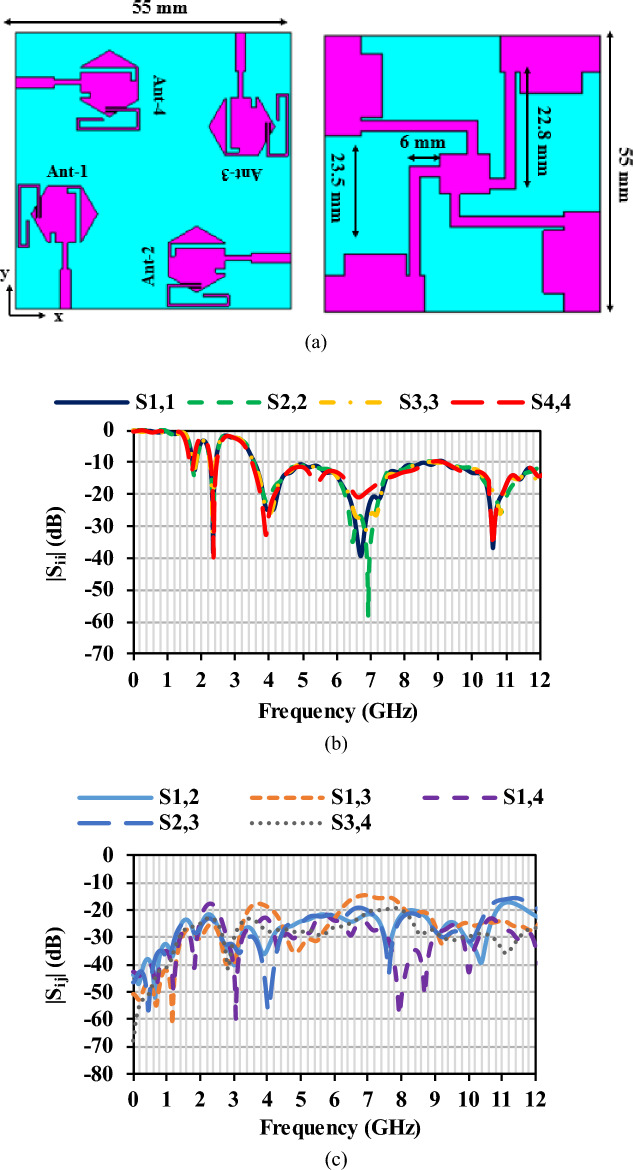
(**a**) Proposed MIMO antenna, (**b**) reflection coefficient characteristics, (**c**) mutual coupling characteristics.

Vehicular applications demand an antenna array as there is a need to receive signals from all directions. The single element shown in Fig. 3 is recreated to four element MIMO antenna, as shown in Fig. 6a. The antenna elements are placed orthogonally to each other to increase isolation and achieve polarization diversity. The dimension of the MIMO antenna is 55 mm × 55 mm × 1.1 mm. The element-to-element spacing is fixed to be 0.059*λ*_0_. The isolation is greater than 15 dB over the operating frequency range. Good isolation is achieved with minimum element spacing. A connected ground structure is used to ensure a common reference voltage. Polarization diversity with dual polarization is achieved when the antennas are placed perpendicular to each other. The measured reflection coefficients and mutual coupling of the MIMO are shown in Fig. 6b and c, respectively. It shows that the antenna has measured mutual coupling lesser than − 15 dB and the reflection coefficients of the MIMO antenna are not affected due to the orientation. The fabrication process of the MIMO antenna is given in the subsequent section.

### Fabrication process of OTA

**Figure 7:**
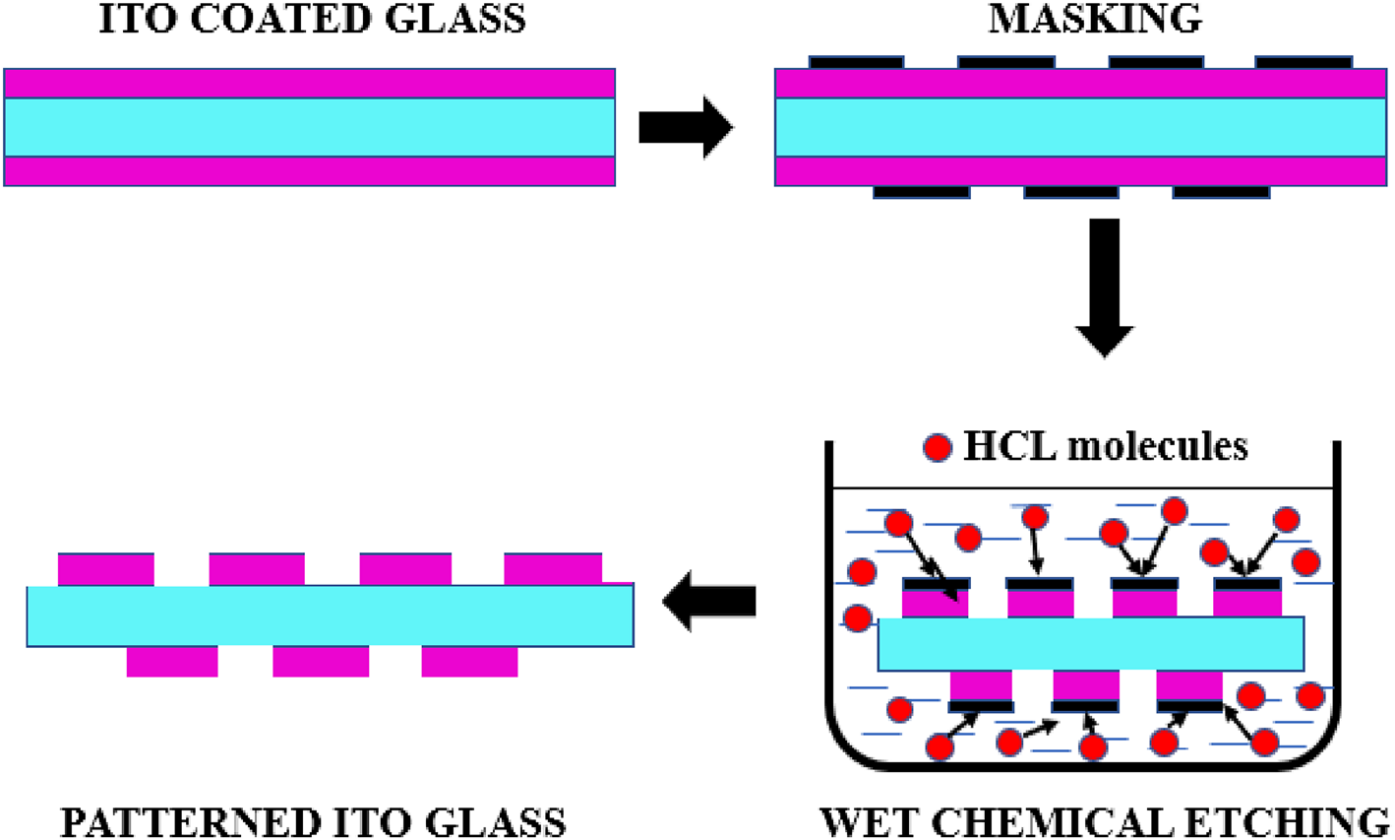
Wet chemical etching process of ITO glass.

**Figure 8:**
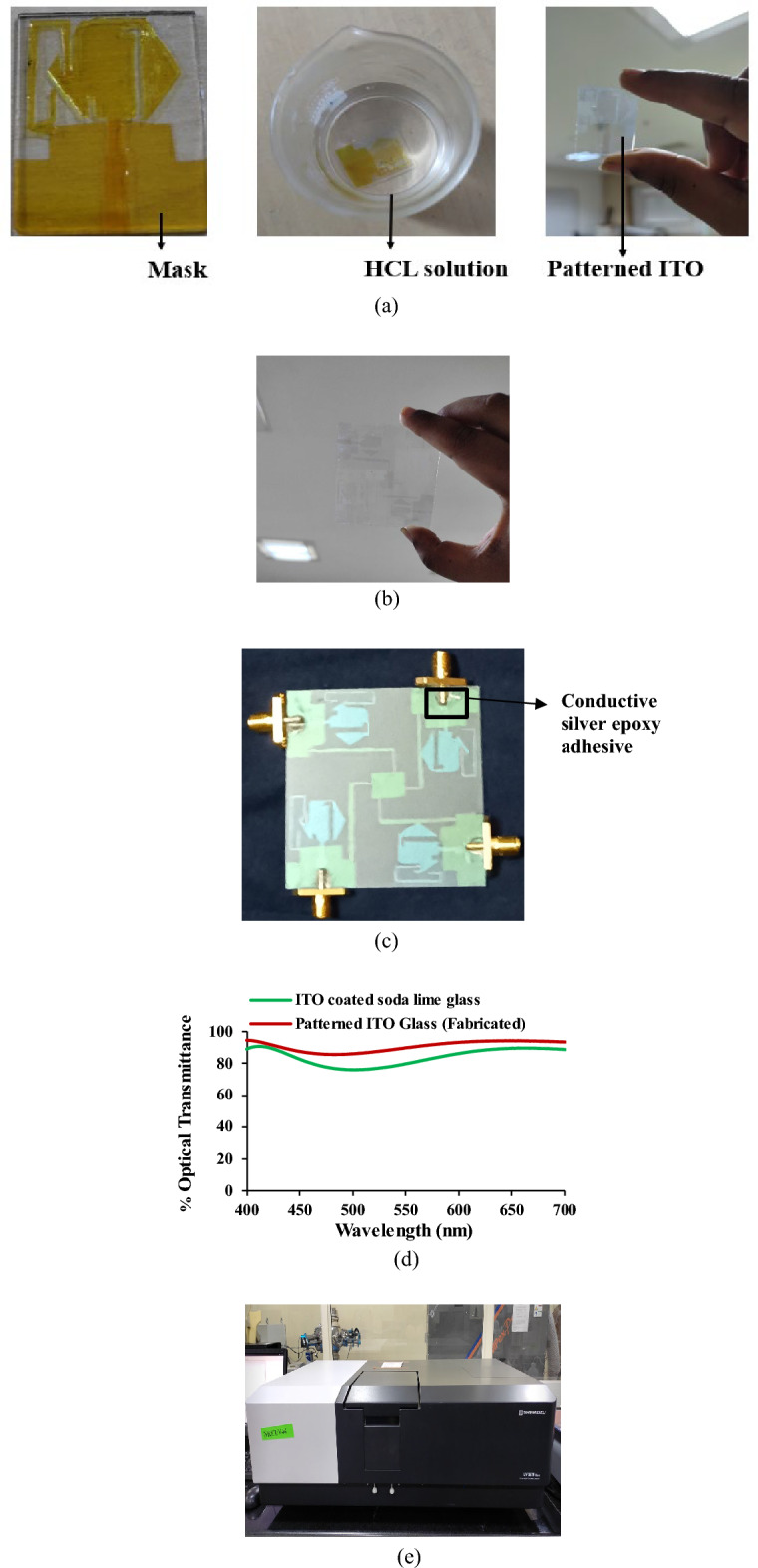
Fabrication process of the OTA (**a**) designed unit under wet chemical etching process, (**b**) patterned MIMO OTA, (**c**) fabricated antenna with connector, (**d**) optical transmittance, (**e**) UV–VIS spectrophotometer.

The soda lime glass of thickness of 1.1 mm coated with ITO on both sides of the glass is used. The thickness of the ITO film is 200 nm. Wet chemical etching is followed to pattern the required antenna design on the substrate. The etching process requires first cleaning the ITO-coated glass with deionized water. Then the required pattern is prepared as a mask using the etch-resistant tape (Kapton tape). The masking is placed on the ITO-coated glass, and the masked ITO is immersed in the HCL: H2O (1:1) solution. The setup is undisturbed for a few minutes. The etching rate varies with the change in HCL concentration. The HCL molecules remove the ITO from the unmasked area, and the required pattern is obtained in the ITO glass. After this process, the patterned glass is exposed to sunlight for about 1 min and cleaned with deionized water. Since hot soldering can melt the layer of ITO material in the substrate, conductive silver epoxy adhesive is used to solder the connector to the antenna. The wet chemical etching of the patterned ITO glass is shown in Fig. 7. The standard ITO glass and the proposed design patterned ITO glass is subjected to testing under the UV-3600 Plus Shimadu spectrophotometer. The results imply that the proposed antenna has a transmittance of greater than 85%. The steps for the fabrication process of the OTA are illustrated in Fig. 8.

## Results and discussion

### Radiation characteristics of the antenna

**Figure 9:**
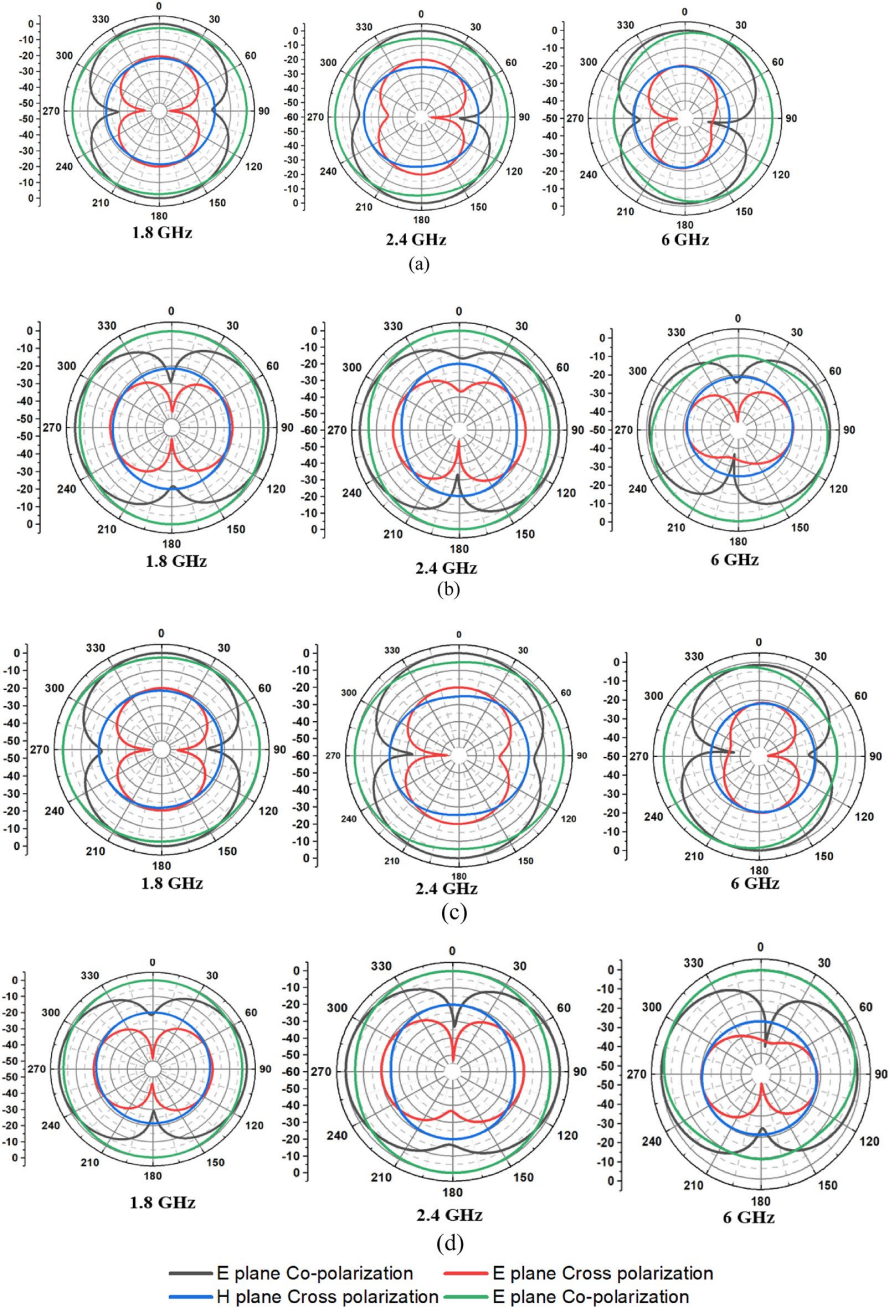
Radiation patterns of the antenna (**a**) Antenna 1, (**b**) Antenna 2, (**c**) Antenna 3, (**d**) Antenna 4.

**Figure 10:**
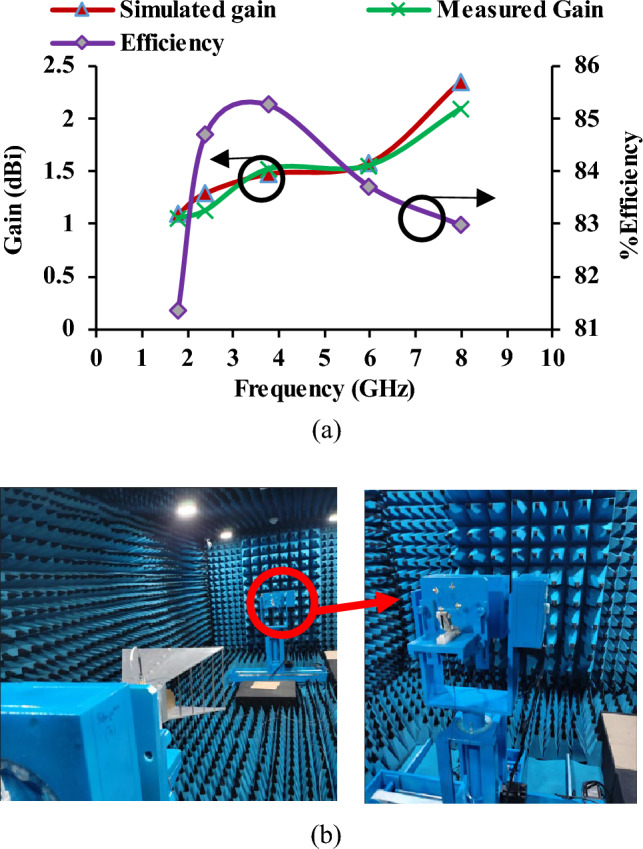
(**a**) Gain and efficiency of the antenna, (**b**) Antenna measurements in an anechoic chamber.

Figure 9 shows the radiation characteristics of the antenna in the E-plane and H-plane. The fabricated antenna prototype is tested in the anechoic chamber. The gain of the antenna is found to be 1.053 dBi, 1.123 dBi, 1.521 dBi, 1.552 dBi, 2.0942 dBi at frequencies of 1.8 GHz, 2.4 GHz, 3 GHz, 6 GHz, and 8 GHz, respectively, and corresponding percentage efficiency is found to be 81.34%, 84.7%, 85.26%, 83.71%, and 82.98%. The gain and efficiency plots are shown in Fig. 10.

### Diversity characteristics

**Figure 11:**
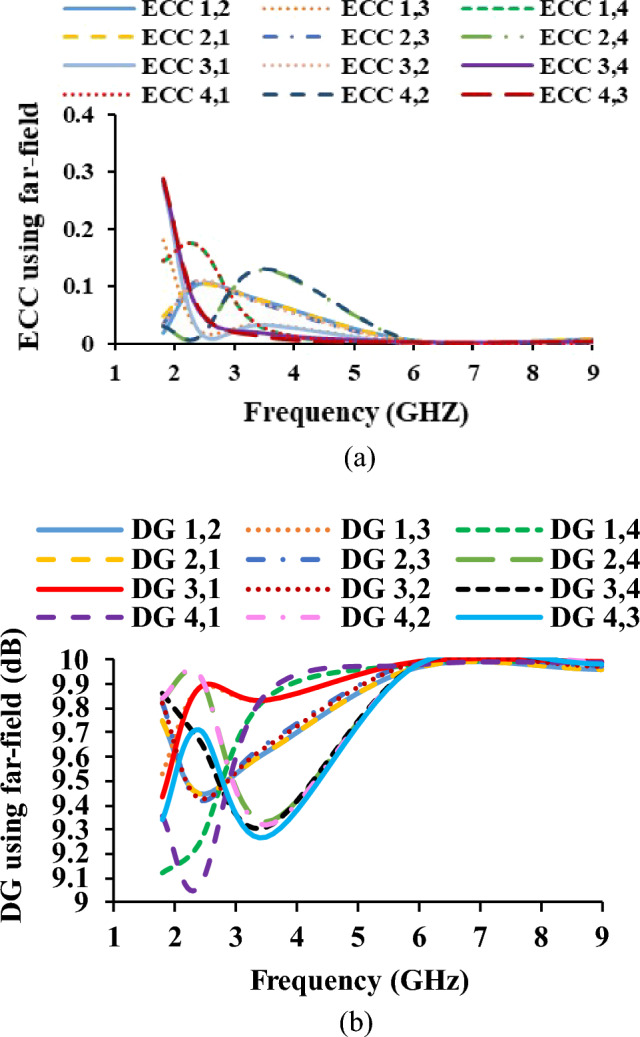
Diversity parameters of the antenna (**a**) ECC, (**b**) DG.

To evaluate the performance of the MIMO antenna, the diversity parameters such as envelope correlation coefficient (ECC), diversity gain (DG), total active reflection coefficient (TARC), and channel capacity loss (CCL) are calculated. ECC is a crucial performance parameter that assesses the effectiveness of diversity in the MIMO system^21,22^. The ECC is calculated using far-field and S-parameter, as given in Eqs. (5) and (6), respectively, which is valid for highly efficient antennas. Figure 11 shows that the ECC over the required frequency bands is less than 0.35.

5 $$ ECC = \frac{{\left| {S_{ii}^{*} S_{ij} + S_{ji}^{*} S_{jj} } \right|}}{{\left( {1 - \left| {S_{ii} } \right|^{2} - \left| {S_{ij} } \right|^{2} } \right)\left( {1 - \left| {S_{ji} } \right|^{2} - \left| {S_{ii} } \right|^{2} } \right)}} $$

6 $$ ECC = \frac{{\left| \rm {{\iint }\left[ {\overrightarrow {{F_{1} }} \left( {\theta ,\varphi } \right) \cdot \overrightarrow {{F_{2} }} \left( {\theta ,\varphi } \right)} \right]d\Omega } \right|^{2} }}{{\rm{\iint }\left| {\overrightarrow {{F_{1} }} \left( {\theta ,\varphi } \right)} \right|^{2} d\Omega \rm{\iint }\left| {\overrightarrow {{F_{2} }} \left( {\theta ,\varphi } \right)} \right|^{2} d\Omega }} $$

**Figure 12:**
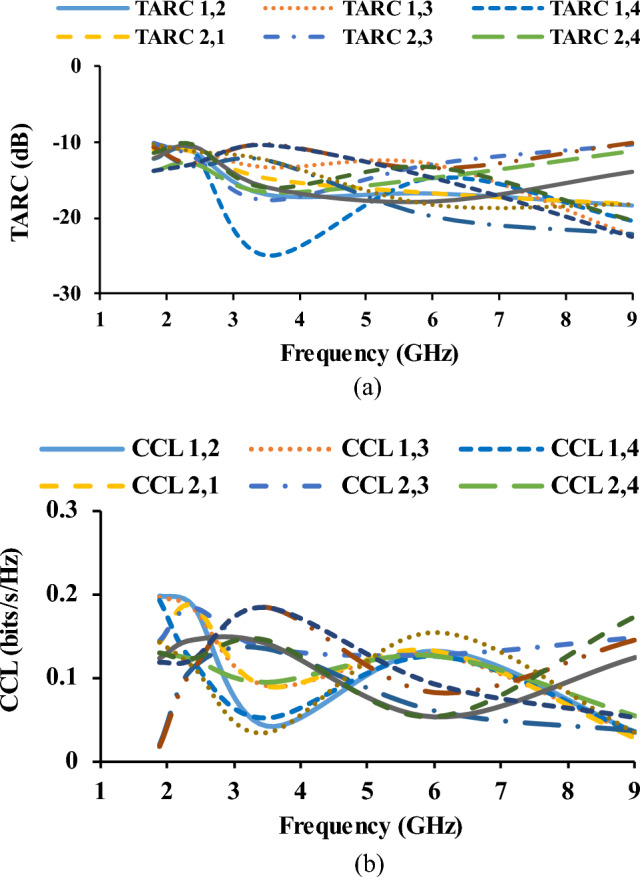
Diversity parameters of the antenna (**a**) TARC, (**b**) CCL.

The DG is calculated using the ECC (far-field and S-parameter)^23,24^. Equation (7) shows the formula for calculating the DG and the corresponding plot is shown in Fig. 12.

7 $$ DG = 10\sqrt {1 - \left| {ECC} \right|^{2} } $$

8 $$ TARC = \frac{{\sqrt {\mathop \sum \nolimits_{i = 1}^{N} \left| {b_{i} } \right|^{2} } }}{{\sqrt {\mathop \sum \nolimits_{i = 1}^{N} \left| {a_{i} } \right|^{2} } }} $$

9 $$ CCL = - log_{2} \left| {\Psi^{R} } \right| $$

TARC is defined as the square root of the total reflected power divided by the total power incident^25^. TARC is calculated using Eq. (8). The CCL provides details regarding the channel capacity loss that occurred in the system while the correlation effect was being used^26^. CCL is calculated using Eq. (9). Figure 12 shows the TARC and CCL plots.

### Vehicular performance of the antenna

**Figure 13:**
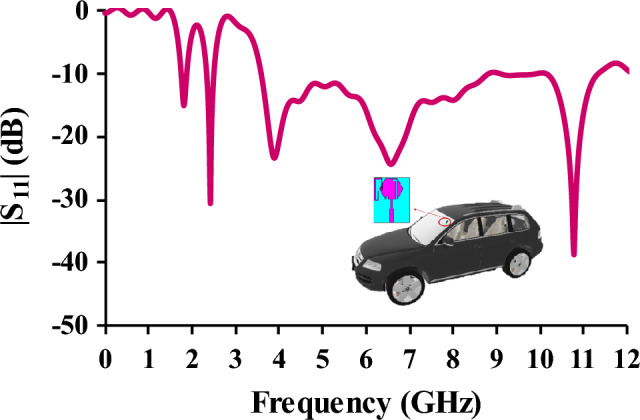
Reflection coefficients in relation to antenna placement in the windshield.

An open-source car model of Volkswagen Touareg^27^ is used to study the performance of the antenna after installation in the windshield of the car (Fig. 13). From the S-parameter analysis, it is identified that the antenna placement on the windshield doesn’t affect the performance.

**Figure 14:**
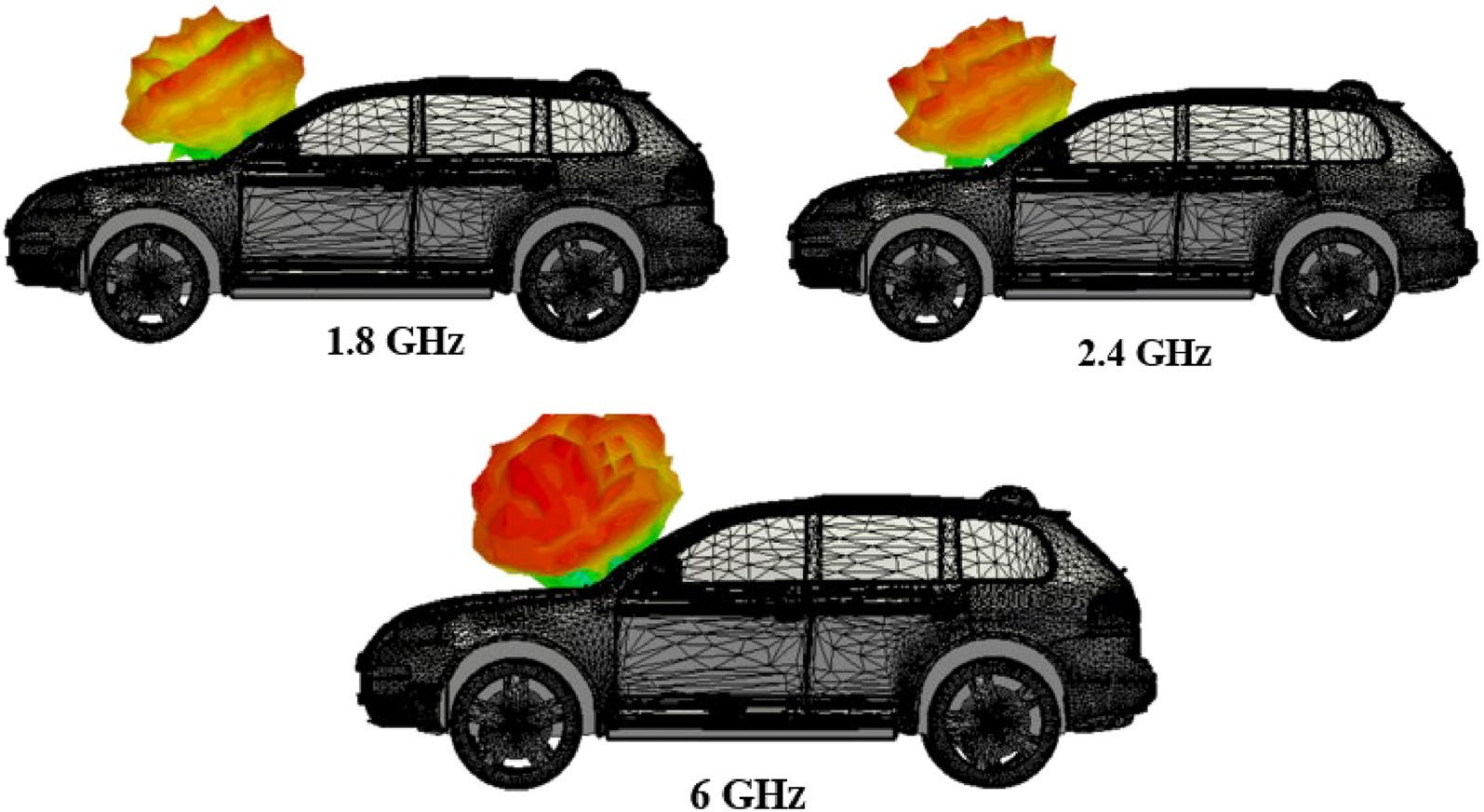
On-car performance of the antenna.

The on-car performance of the antenna is studied (Fig. 14) by placing the prototype in the windshield of the imported 3-D car model. The study is proposed for 1.8 GHz, 2.4 GHz, and 6 GHz frequencies. The study shows that the antenna placed over the vehicle offers omnidirectional characteristics. The directivity values at 1.8 GHz, 2.4 GHz, and 6 GHz are 7.79 dBi, 7.18 dBi, and 6.64 dBi, respectively.

**Figure 15:**
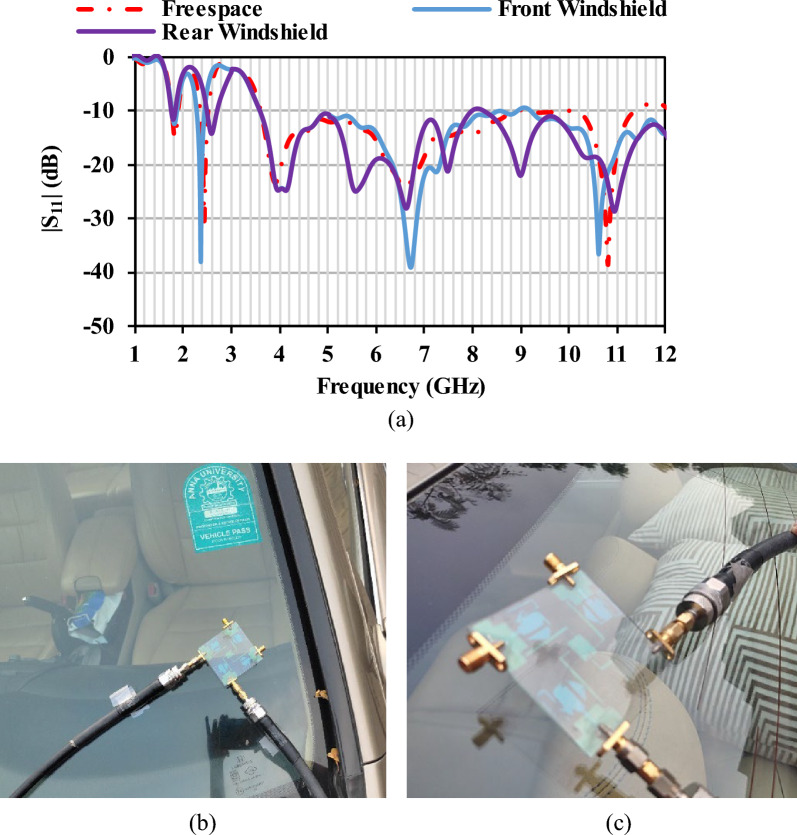
(**a**) Reflection coefficients of the antenna when it is mounted on the car windshield, (**b**) antenna placement on the front windshield glass of the car, (**c**) antenna placement on the rear windshield glass of the car.

The proposed optically transparent antenna is placed in the front windshield and rear windshield for studying the antenna impedance characteristics. The reflection coefficient characteristics in Fig. 15 shows that the antenna performance is not highly affected after the placement on the windshield. The reflections from the ground and the metal plate/surrounding objects are the uncertainties that creates slight variation in the impedance characteristics.

The salient features of the proposed MIMO OTA antenna are given in the Table 1.

- The proposed unit cell antenna has overall dimension of 0.33*λ*_0_ × 0.33*λ*_0_, which is smaller than the other works in the literature^23,26,27,31,32,34^.
- The proposed antenna is developed suing the soda lime glass substrate with ITO coating. The substrate is affordable and easily available than other materials reported in the literature.
- The designed MIMO antenna works for the tri-band frequencies covering 1.8 GHz, 2.4 GHz, and 3.39–12 GHz, whereas other works reported in the literature^23–34^ offers single-band or dual-band operation.
- The gain of the antenna is observed to be greater than 1 dBi and efficiency found to be greater than 80%.
- The designed MIMO antenna consists of four elements placed orthogonally and offers dual polarization, unlike other antennas reported in the literature^23–34^.
- The proposed OTA is fabricated using the wet chemical etching process, which is inexpensive and also easy for fabrication, unlike other works reported in the literature^23–33^.

**Table 1:** Comparison of the proposed antenna to previous literature.

Refs.	Size (*λ*_0_ × *λ*_0_)	Substrate	Conductive surface	Frequency (GHz)	Number of operating bands	Gain (dBi)	Efficiency (%)	No of Elements	Fabrication Type
^15^	8.54 × 8.54	Plexiglass	AgHT-8	2.44, 3.7	2*	3.6, 7.1	74, 84	2	Pasting of thin film
^16^	0.312 × 0.4	Soda lime glass	FTO/ITO	2.4–11	1**	2	60	2	Sputtering and etching
^18^	0.63 × 059	Soda lime	FTO	3–11	1**	3.2	50	1	Sputtering
^19^	0.326 × 0.571	Soda lime	FTO	4.9	1*	5.14	90	1	Sputtering
^28^	0.5 × 0.6	PET	AgHT-8	3–13	1**	−2	20	1	Pasting of thin film
^29^	0.079 × 0.095	Plexiglass	AgHT-8	23.92–43.98	1**	1.94	> 85	1	Pasting of thin film
^30^	0.245 × 0.245	Borosilicate glass	AgHT-8	2.45, 5.26	2*	0.64, 1.2	62, 83	1	Pasting of thin film
^31^	0.75 × 1.01	PET	AgHT-8	3.89–5.97	1**	3	80	1	–-
^32^	0.28 × 0.28	Plexiglass	AgHT-8	2.4, 5.5	2*	0.7, 1.67	–-	1	Pasting of thin film
^33^	0.208 × 0.192	PET	AgHT-8	1.6–2.95, 5.4–6.4	2**	−3.25, −4.53	> 80	1	Pasting of thin film
^34^	0.166 × 0.166	Glass	ITO	1–7	1**	5	25	1	Sputtering
^35^	0.32 × 0.32	Glass	MMMC	2.4–2.8, 5.15–5.8	1*, 1**	0.74, 2.3	40	1	Pasting of thin film
Prop.	0.33 × 0.33	Soda lime glass	ITO	1.8, 2.4, 3.39–12	2*, 1**	2.049	> 80	4	Wet chemical etching

*Narrowband, **Wideband.

The MIMO performance characteristics are compared in Table 2.

1. The antenna has smaller dimensions in comparison with the other antennas reported in the literature^15,16^ and^36^.
2. The proposed MIMO antenna has four elements placed orthogonally to each other.
3. The antenna provides ECC of less than 0.3, DG of greater than 9 dB, CCL less than 0.2 bits/s/Hz, and TARC less than – 10 dB.
4. Unlike other MIMO antennas, the proposed MIMO antenna provides dual polarization with two polarization vectors due to orthogonal orientation of the antenna elements.

**Table 2:** MIMO antenna performance comparison.

Refs.	Size (*λ*_0_ × *λ*_0_)	No of Ports	ECC	DG (dB)	CCL (bits/s/Hz)	TARC (dB)	Polarization
^15^	8.54 × 8.54	2	0.002	> 9.9	–-	–-	Horizontal
^16^	0.312 × 0.4	2	< 0.4	> 9.5	–-	–-	Horizontal
^36^	1.92 × 1.6	4	< 0.1	> 9.5	–-	< -15	Horizontal
Prop.	0.33 × 0.33	4	< 0.3	> 9	< 0.2	< -10	Dual (Horizontal and Vertical)

### Machine learning algorithm for vehicular classification

Over the past ten years, there has been an increase in research into and usage of machine learning approaches to address the issues of malware identification and categorization. Obtaining accessible data, cleaning and preparing the data, constructing models, testing the models, and putting the models into production are the steps in the iterative machine learning workflow process^37–46^.

In this paper, machine learning algorithm is used for classification and identification of the vehicles in the scenarios like parking, toll collection, and unnecessary intrusion. This paves the platform for the various vehicular applications like intelligent transport system (ITS), electronic toll collection (ETC), parking management, fleet management, and advanced driving assisting system (ADAS). For the classification of vehicles, two input parameters are used: frequency and reflection coefficients. The reflection coefficients of different vehicles (auto (three wheeler), car, and truck) are obtained by placing the antenna in the corresponding vehicle. The machine learning method reduces the dimensionality of the features using linear discriminant analysis (LDA). The trained multilayer perceptron (MLP) has given the projected feature obtained from the LDA. The system performance is tested for the vehicle classification using the MLP classifier. The required training set is obtained by placing the antenna in the windshield of vehicle, and it is tested on the set of values obtained by placing the antenna in the sidemirror of the vehicles. For classification, maximum sample size of 30,000 and minimum of 3000 is chosen (see Table 3). An accuracy of 80% is obtained for a minimum window size of 3000.

**Table 3:** Antenna classification accuracy in different scenarios.

Vehicle scenarios	Sample window size	Antenna classification accuracy
Idle (Parking lot)	30,000	88.14
	27,000	87.93
	24,000	87.2
Electronic Toll Collection	21,000	85.4
	18,000	85.93
	15,000	84.8
	12,000	84.32
Traffic Intrusion	9000	83.3
	6000	82.91
	3000	80.4

A concept from machine learning called a confusion matrix contains data on both actual and anticipated classifications made by a classification system^47^. The columns of a confusion matrix are normally the same categories as those established by a ground survey, and the cell values are the number of observations attributed to each grouping of categories. One dimension of a confusion matrix is indexed by the actual class of an object, and the other is indexed by the predicted class of the classifier. The confusion matrix for the vehicle classification is shown in Table 4. The confusion matrix is developed with 20 samples for each set. The number of samples in the diagonal column shows the correctly identified samples. The dark-shaded cell along the diagonal represents the correctly identified reflection coefficients, and the light-shaded cell shows the wrongly identified reflection coefficients.

**Table 4:**
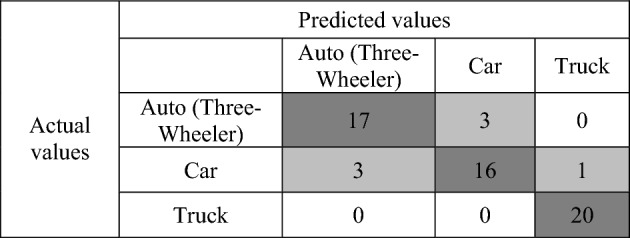
Confusion matrix for vehicle identification.

To determine a classification model's performance metrics, the confusion matrix is analyzed with some metrics. Precision describes the percentage of all favorable predictions that come true. Precision is the proportion of recovered instances that are relevant, also known as positive predictive value.

10 $$ Precision = \frac{TP}{{TP + FP}} $$

Sensitivity, probability of detection, and true positive rate are other names for recall. It relates to the percentage of real positives that are appropriately identified.

11 $$ Recall = \frac{TP}{{TP + FN}} $$

Specificity, often known as the real negative rate, is the percentage of actual negatives that are correctly identified as such. Recall's opposite is specificity.

12 $$ Specificity = \frac{TN}{{TN + FP}} $$

The accuracy of a test is assessed using the F1 score (see Table 5), which is the harmonic mean of precision and recall. A minimum score of 0 and a maximum score of 1 are both possible (perfect recall and precision). It is an estimation of the model's overall accuracy and dependability.

13 $$ F1_{score} = \frac{{2*\left( {Prediction*Recall} \right)}}{Prediction + Recall} = \frac{2TP}{{2TP + FP + FN}} $$

**Table 5:** Confusion matrix metrics for vehicle classification.

	Precision	Recall	Specificity	F1 Score	Negative Prediction Value
Auto (Three-Wheeler)	85	85	92.5	85	92.5
Car	84.2	80	92.5	82	90.2
Truck	92.23	100	97.5	97.5	100

The negative predictive value in machine learning is the proportion of anticipated negatives that actually happen.

14 $$ Negative \;Prediction Value = \frac{TN}{{TN + FN}} $$

## Conclusion

The proposed OTA antenna operates for tri-band frequencies covering 1.8 GHz, 2.4 GHz, and 3.39–12 GHz for GSM, Bluetooth, Wi-Fi, and vehicle to vehicle communication applications. The transparent antenna is developed using the wet chemical etching process. The MIMO antenna with four elements placed orthogonally provides dual polarization. The antenna prototype is tested in the anechoic chamber and the gain and efficiency are found to be greater than 1 dBi and 80%, respectively. The diversity parameter values are within the acceptable limit. The antenna prototype is mounted in the windshield of a vehicle and study shows that there is no effect on the antenna performance. Machine learning for the classification of vehicles in different scenarios is proposed, and required parameters are studied, which provide classification accuracy of 80% for minimum window size. Hence, the proposed antenna is suitable for vehicular applications.

## Data Availability

The datasets used and analyzed during the current study are available from the corresponding author on reasonable request.
